# A trend analysis and sub-regional distribution in number of people living with HIV and dying with TB in Africa, 1991 to 2006

**DOI:** 10.1186/1476-072X-8-65

**Published:** 2009-11-24

**Authors:** Olalekan A Uthman, Ismail Yahaya, Khalid Ashfaq, Mubashir B Uthman

**Affiliations:** 1 Department of Public Health, Epidemiology and Biostatistics, University of Birmingham, Edgbaston, Birmingham, B15 2TT, UK; 2 Department of Epidemiology and Community Health, University of Ilorin, Ilorin, Kwara State, Nigeria

## Abstract

**Background:**

The tuberculosis (TB) bacillus and the Human Immunodeficiency Virus (HIV) have formed a powerful alliance and are together responsible for more than five million deaths per year. TB is leading to increased mortality rates among people living with HIV/acquired immunodeficiency syndrome (AIDS). The aim of this study was to investigate the geographical and temporal distribution of TB-HIV deaths in Africa in order to identify possible high-risk areas.

**Methods:**

Time trends in the 16-year study period from 1990 to 2005 were analyzed by multilevel Poisson growth curve models. Moran global and local indicators of spatial associations were used to test for evidence of global and local spatial clustering respectively.

**Results:**

Eastern, Southern, Western, and Middle Africa experienced an upward trend in the number of reported TB-HIV deaths. The spatial distribution of TB cases was non-random and clustered, with a Moran's I = 0.454 (p = .001). Spatial clustering suggested that 13 countries were at increased risk of TB-HIV deaths, and six countries could be grouped as "hot spots".

**Conclusion:**

Evidence shows that there is no decline in growth in the number of deaths due to TB among HIV positive in most Africa countries. There is presence of 'hot-spots' and very large differences persist between sub-regions. Only by tackling TB and HIV together will progress be made in reversing the burden of both diseases. There is a great need for scale-up of preventive interventions such as the World Health Organization '3I's strategy' (intensified case finding, isoniazid preventive therapy and infection control).

## Background

From the early stages of the Human Immunodeficiency Virus (HIV) epidemic, a strong association with tuberculosis (TB) was apparent [1] and HIV subsequently emerged as one key factor undermining global TB control [2]. Despite being preventable and treatable, TB remains the most common life-threatening opportunistic infection and a leading cause of deaths among people living with HIV/AIDS (PLWHA) [3]. In Africa, which has the highest rates of both diseases, TB is the leading killer of PLWHA [2,4-7]. Deaths from TB among HIV-positive people (henceforth referred to as TB-HIV deaths) have exacted a huge toll on the worst affected communities in sub-Saharan Africa [3]. In recent years, the excess HIV-TB mortality has been 1.6-fold greater in women compared with men [3]. Unprecedented resources have been invested in HIV/AIDS throughout low-income countries, but without a concomitant scale-up in basic TB control and without a concerted response to TB and HIV co-infection. TB now threatens the successes borne out of that investment. All advances in the treatment of HIV and TB to date have the potential to be undermined, or worse, reversed, by the widespread failure to respond to the dual TB/HIV co-epidemic in a coordinated and integrated way.

The challenge facing the TB and HIV control programmes in Africa is that the disease burden is not homogenous but varies geographically. Minimizing the risk of TB-HIV deaths can be assisted by recognizing its geographical distribution and identifying areas of high risk. Policy makers and researchers often want to know the distribution of a disease incidence by geographical region or associated environmental factors [8]. The ability to map spatial and temporal variation in disease risk is more important than ever, given the ever-increasing disease burden in Africa[9]. In this regard, mapping and investigating risk variations in TB-HIV deaths is an invaluable tool. Furthermore, mapping the variation in risk can help improve the targeting of scarce resources for public health interventions. This information is useful both for (1) micro-targeting future programs and (2) identifying locales where progress has been made to investigate interventional, socio-economic, and cultural conditions that may have contributed to the apparent progress. In the context of Sub-Saharan Africa countries, most research has been largely limited to trend analysis of prevalence of HIV and incidence of TB. To the best of our knowledge, there has been no study performed to date that examined the trend and geographical disparities in TB-HIV deaths in sub-Saharan Africa. Without objective information about the current patterns of TB-HIV deaths, it is difficult to plan substantial public health programs that could prevent and lead to reduction in TB-HIV deaths. With the above issues in mind, the objective of this study was to describe the geographical and temporal distribution of TB-HIV deaths in Africa in order to identify countries with unusually high rates.

## Methods

### Data

This study primarily uses data from the World Health Organization (WHO) statistical information system to describe the temporal and spatial variations in TB-HIV deaths in Africa. The data set includes the estimated deaths due to TB among HIV positive people reported between 1991 and 2006 (expressed as rate per 100,000 populations). Estimates are based on annual case notifications, on special surveys of the prevalence of infection or disease and on information from deaths (vital) registration systems. All forms of TB were included. Estimates of mortality are based on a consultative and analytical process and are published annually [10]. Available data differ from country to country but include case notifications and death records (from routine surveillance and vital registration). For further details the readers may go through the published literature [2,10,11].

### Africa Sub-regions

For this study, Africa was divided into five sub-regions based on a combination of geographic, economic and scientific criteria. The sub-regions are Northern Africa, Western Africa, Middle Africa, Eastern Africa and Southern Africa.

### Estimation of Time Trend

Temporal patterns were displayed by plotting yearly TB-HIV deaths against year. Time trends in the 16-year study period from 1991 to 2006 were analyzed by multilevel Poisson growth curve models. In this two-level model, TB-HIV deaths for each calendar year referred to level-1 and the level-2 unit as each country. Time-on-study was modelled as four different time periods, allowing time effects to be nonlinear. The quartiles were 1991 to 1994 (reference), 1995 to 1998, 1999 to 2002, and 2003 to 2006. Time trend may differ across sub-regions. The time-trend analysis was done in two steps. In the first approach, all five sub-regions were pooled into one datum set. In the second model, all five sub-regions were analysed separately.

### GIS mapping and Smoothing

For conducting a GIS (geographic information system)-based analysis on the spatial distribution of TB-HIV deaths, the country-level polygon map was obtained, on which the country-level point layer containing information regarding latitudes and longitudes of central points of each county was created. All TB-HIV deaths were geocoded and matched to the country-level layers of polygon and point by administrative code. To alleviate variations of number of TB-HIV deaths in small populations and areas, annualized average TB-HIV deaths per 100,000 at each administrative region over the 16-year period were calculated [12]. Spatial rate smoothing was carried out using empirical Bayes approach. Smoothing geographically-defined data can uncover unexpected features, patterns, or gradients that one might not otherwise detect from a display [13,14]. Geographically-defined data are often amenable to smoothing, since data in one region are likely affected, to a greater or lesser extent, by data in neighbouring regions [13,14]. Smoothing borrows neighbouring information in a flexible way to permit exploratory analyses and provide indications of possible patterns that one might otherwise find difficult to detect [13-16]. In addition, smoothing can reduce attention to unusual values or outliers [13-16]. Based on annualized average incidence, all countries were grouped into quartiles-based categories: non-endemic area (1st quartile); low-endemic area (2nd quartile); medium-endemic area (3rd quartile); and high-endemic area (4th quartile). The four categories of country were colour coded on maps. To assess the risk of TB-HIV deaths in each country, an excess hazard map was produced. The map represents the ratio of the observed number of TB-HIV deaths for each country over the expected number of TB-HIV deaths. A likelihood function was used to test for elevated risk within the country in comparison with risk outside the country. The likelihood function for any given country was proportional to:(1)

where *D *is the total number of TB-HIV deaths, *d *is the number of TB-HIV deaths within the country and *n *is the expected number of TB-HIV deaths. The indicator function I() is 1 when TB-HIV deaths in the country are more than expected; otherwise it is 0. The excess risk is a non-spatial measure, which ignores the influence of spatial autocorrelation [12].

### Spatial Autocorrelation Analysis

The Global Moran's *I *[17] was used as a measure of the overall clustering and is assessed by a test of a null hypotheses. Rejection of this null hypothesis suggests a spatial pattern or spatial structure. Spatial connectivity was incorporated by a spatial weights matrix "W" [17]. The global measure of Moran's *I *is defined as:(2)

where *w*_*ij *_is the row-standardized contiguity matrix, *x*_*i *_is the risk scale measure at country *I, *and *x*_*j *_is the risk scale measure at country *j*, and *μ *is the average level of risk.

While Moran's *I *provides a measure of the overall clustering, it does not show "where the clusters or outliers are located, nor what type of spatial correlation is most important (e.g. correlation between high or between low values)" [18].

Local measures of spatial association provide a measure of association for each unit and help identify the type of spatial correlation--these are carried out using the Local Indicators of Spatial Association (LISA) [17]. The Local Moran's *I *is used as an indicator of local spatial association. The local measure of Moran's I is defined as:(3)

The Moran significance map builds on the Moran scatter plot and incorporates information about the significance of "local" spatial patterns. The Moran scatter plot helps identify the nature of spatial autocorrelation between countries so they can be categorised into four groups. For the TB-HIV deaths these are:

• *High-high*: high value of TB-HIV deaths in a country, neighbouring countries have high values of TB-HIV deaths (spatial clusters).

• *Low-high*: low value of TB-HIV deaths in a country, neighbouring countries have high values of TB-HIV deaths (spatial outliers).

• *Low-low*: low value of TB-HIV deaths in a country, neighbouring countries have low values of TB-HIV deaths (spatial clusters).

• *High-low*: high value of TB-HIV deaths in a country, neighbouring countries have low values of TB-HIV deaths (spatial outliers).

After computing the appropriate statistic from the smoothed rates, a Monte Carlo Randomization (MCR) procedure was used to recalculate the statistic from the randomized data observations to generate a reference distribution using 999 permutations. The p-values were computed by comparing the observed statistic to the distribution generated by the MCR process and significance level was set as .001.

### Software

Exploratory spatial data analysis (ESDA) was carried out through the GeoDa software[19]. GeoDa provides a very user-friendly environment to implement ESDA methods and is freely downloadable [20]. Stata versus 10 for Windows was used multilevel Poisson growth curve models (Stata Corp., College Station, TX, USA).

## Results

### Temporal variability

**Figure 1:**
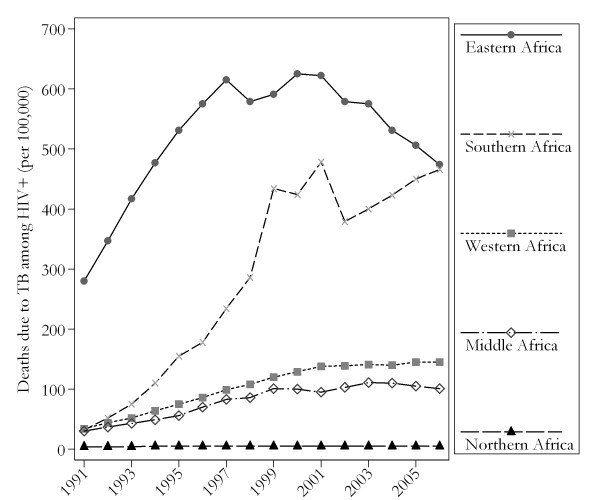
**Temporal trends in HIV-TB deaths, African sub-regions, 1991 to 2006**.

Figure 1 depicts the trends of reported TB-HIV deaths in the period 1991 to 2006. Eastern Africa ranked first in absolute upward trend in number of reported TB-HIV deaths, followed by Southern and Western Africa. Growth curve analysis confirmed a continual increase in the number of reported TB-HIV deaths in all African sub-regions except for Northern Africa (Table 1). Compared to the reference period (1991 to 1994), incidence risk ratios (95% confidence interval) in 2003 to 2006 were 6.46(5.69 to 7.35), 2.94(2.50 to 3.46), 2.69(2.24 to 3.22), and 1.37(1.28 to 1.47) for Southern, Western, Middle, and Eastern Africa, respectively. This equates to a total increase of 546%, 194%, 169%, and 37% in the TB-HIV deaths between 1991 - 1994 and 2003-2006. The time-trend analysis was not significant for Northern Africa.

**Table 1:** Temporal trends in TB cases by sub-regions in Africa, 1991 to 2006

	Incidence rate ratio*(95% CI)			
	
	1991-1994	1995-1998	1999-20002	2003-2006^p-trend^
**All Africa**	1 (reference)	1.78(1.68 to 1.87)	2.35(2.24 to 2.47)	2.24(2.13 to 2.36)^.000^
**Sub-regions**				
Eastern Africa	1 (reference)	1.51(1.42 to 1.61)	1.59(1.49 to 1.69)	1.37(1.28 to 1.47)^.000^
Western Africa	1 (reference)	1.90(1.59 to 2.26)	2.71(1.30 to 3.20)	2.94(2.50 to 3.46)^.000^
Southern Africa	1 (reference)	3.17(2.76 to 3.63)	6.38(5.61 to 7.25)	6.46(5.69 to 7.35)^.000^
Middle Africa	1 (reference)	1.86(1.53 to 2.50)	2.51(2.09 to 3.02)	2.69(2.24 to 3.22)^.000^
Northern Africa	1 (reference)	1.18(0.62 to 2.25)	1.18(0.62 to 2.25)	1.18(0.62 to 2.25)^.950^

*estimates are from Multilevel Poisson growth curve models

### Spatial Distribution and Autocorrelation

**Figure 2:**
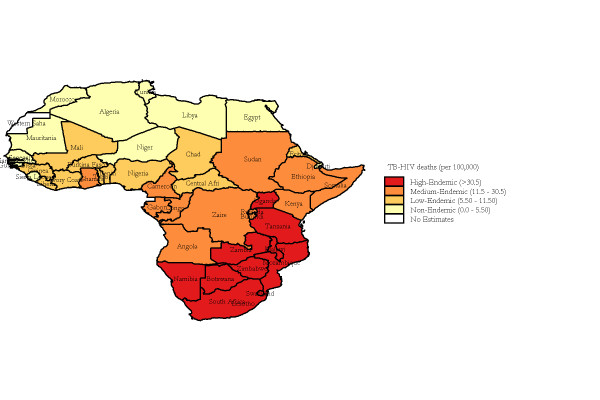
**Spatially smoothed percentile map of annualized average TB-HIV deaths in Africa, 1991 to 2006**.

**Figure 3:**
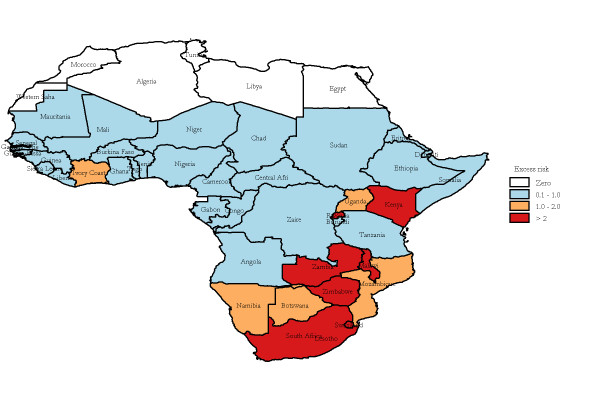
**Excess hazard map of annualized average TB-HIV deaths in Africa, 1991 to 2006**.

**Figure 4:**
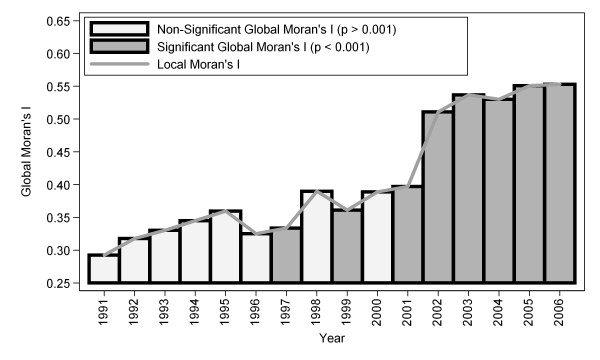
**Global and local spatial autocorrelation analyses for TB-HIV deaths in Africa, 1991 to 2006**.

**Figure 5:**
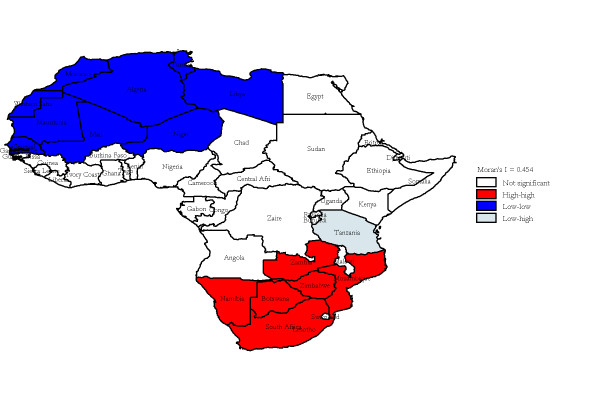
**Local Indicator of Spatial Association (LISA) cluster map for annualized average TB-HIV deaths in Africa, 1991 to 2006**.

The median annualized average TB-HIV deaths at the country level was 8.88 (range: 0 to 113.25) per 100,000 population per year. Twelve countries were non-endemic, with annualized average TB-HIV deaths between zero and 5.50 per 100,000; 12 were low-endemic, with incidence between 5.50 and 11.50 per 100,000; 12 were medium-endemic, with incidence between 11.50 and 30.50 per 100,000; and 11 were high-endemic, with incidence greater than 30.50 per 100,000. The four types of areas were displayed in the thematic map as shown in Figure 2. A spatially smoothed percentile map of annualized average TB-HIV deaths was created; smoothed rate presents a better pattern and shows clearly where the problem was most severe (Figure 2). The excess hazard map depicted in Figure 3 showed distribution of the excess risk, which was a ratio of the observed number over the expected number of cases. Countries marked in blue had lower incidences than expected, as showed by excess risk values less than 1. In contrast, countries in red and light yellow had incidences higher than expected, with risk values greater than 1. Figure 4 shows results of global spatial autocorrelation analyses for TB-HIV deaths in Africa from 1991 to 2006. As shown in figure 4, the value of Global Moran's I increased from 0.293 in 1991 to 0.553 in 2006. The formal test of spatial dependence was not significant from 1991 to 1996 (p > 0.01). The formal test were significant at p < .001 for 2001 to 2006 and annualized average. Thus, we rejected the null hypothesis of spatial independence and concluded that there was sufficient evidence of spatial dependence. The results of Local Moran's I show statistically significance spatial autocorrelation (Moran's I = 0.454, *p *= .001) (Figure 5). Southern and some parts of Eastern Africa belong to High-high (hot-spot) clusters. These are locations with high TB-HIV deaths with similar neighbours. The locations marked in blue belong to Low-low (cold-spot) clusters. These are countries with low TB-HIV deaths with similar neighbours. The United Republic of Tanzania is the only country in a Low-high cluster, potential an outlier. In order words, the United Republic of the Tanzania is a country with low TB-HIV deaths and with high-incidence neighbours. The other countries marked in white are locations with no statistically significant autocorrelation.

## Discussion

### Temporal variability

We have provided evidence that the number of TB-HIV deaths increased yearly in Africa except for North Africa. The observed trends varied markedly across the African sub-regions, such that Eastern Africa ranked first in absolute upward trend in number of reported TB-HIV deaths, followed by Southern and Western Africa. The rising trend in TB-HIV deaths that we observed echoes findings in a study by Zwang and colleagues[21]. Zwang et al[21] investigated trends and age-sex patterns of mortality in PTB/HIV co-infection in a rural population in South Africa. They found that mortality related to PTB/HIV co-infection began somewhat earlier among men than among women, and increased quickly over time[21]. A similar trend has been reported separately for incidence of TB [2,22-25] and prevalence of HIV in sub-Saharan Africa[26]. Rising levels of TB co-infection with HIV in immigrants from Africa have also been documented in the literature [27,28]. Ahmed and co-researchers [27] examined the growing impact of HIV infection on the epidemiology of TB in England and Wales between 1999 and 2003; and found that about six percent of the cases of TB were co-infected with HIV and there was a yearly increase in the proportion. In addition, the study provided evidence that patients co-infected with HIV were predominantly those born abroad. Among cases of TB of black African origin, women had a higher proportion co-infected with HIV than men. Velasco and co-researchers [28] reported that most TB-HIV infected immigrants in Spain are from Africa.

The unprecedented growth of the TB epidemic in Africa is attributable to several factors, the most important being the HIV pandemic. Driven by a generalized HIV epidemic [2,4,6,7,29] and compounded by weak health care systems, inadequate laboratories, and conditions that promote transmission of infection, this devastating situation has steadily worsened, exacerbated by the emergence of drug-resistant strains of TB. There were an estimated 1.37 million HIV positive TB patients globally in 2007 [10,30]. Around 80% of patients live in sub-Saharan Africa. 456,000 people died of HIV-associated TB in 2007 [10,30]. At least one-third of the 33.2 million people living with HIV worldwide are infected with TB and are 20-30 times more likely to develop TB than those without HIV. Without proper treatment, about 90 percent of PLWHA die within months of developing active TB disease [31]. With the recent spread of drug-resistant TB, this already complicated interplay between TB and HIV has only become more deadly, more costly, and more difficult to address. Because most countries throughout Africa have little or no capacity to test for TB drug resistance, and also due to the increased difficulty of treatment and the already high mortality associated with standard TB-HIV co-infection, drug-resistant TB has resulted in mortality rates exceeding 95 percent in PLWHA in some settings [10,31].

### Spatial variability

In the study, exploratory spatial data analysis (ESDA) methods [17,32] were carried out to study spatial relationship in TB-HIV deaths at country level in Africa. ESDA is an extension of exploratory data analysis (EDA) that focuses on detecting spatial patterns in data and the generation of hypotheses based on the spatial patterns in the data [33,34]. TB-HIV deaths were mapped from different aspects such as crude incidence, excess risk and spatially smoothed incidence. In addition, the study evaluated spatial patterns and highlighted geographic areas with significantly high TB-HIV deaths in Africa. The study showed that the spatial distribution of TB-HIV deaths in Africa was non-random and with evidence of statistically significant clustering. Through exploratory spatial analyses, the study was able to pinpoint geographic areas with higher risk and to assess temporal variability of the risk areas, thus providing a working hypothesis on risk of TB-HIV deaths and environmental exposures. Geographic areas with higher cases of TB need further epidemiologic investigation for potential relationships between lifetime environmental exposures and risk of TB-HIV deaths.

### Policy implications

Despite the link between the two diseases being acknowledged as far back as the 1980s, efforts to control TB and HIV/AIDS remain largely independent of each other, an oversight that has resulted in millions of unnecessary deaths. Only by tackling TB and HIV together will progress be made in reversing the burden of both diseases. All advances in the treatment of HIV/AIDS and TB to date have the potential to be undermined, or worse, reversed, by the widespread failure to respond to the TB/HIV co-epidemic in a coordinated and collaborative way. To accelerate progress towards epidemiological impact targets set for 2015, HIV-TB must move up the global public health agenda with increased resource allocation and concerted international action. There is a great need for scale-up of preventive interventions such as the WHO '3I's strategy'.

To finally stop people living with HIV from dying of TB, there is a need for bold action from all sectors. One such strategy would be to screen all PLWHA accessing HIV/AIDS care for TB and make the "3 Is"--intensified case finding, infection control, and isoniazid preventive therapy--central to HIV/AIDS services and universally available [35]. Affected countries, donors, and technical agencies must join together to craft a plan to ensure universal access to high quality TB-HIV care by the year 2015 - moving in coordination with the goal of universal access on HIV by 2010. Finding and treating TB cases, administering antiretroviral therapy (ART) and isoniazid preventive therapy (IPT), and infection control are critical activities to incorporate into HIV care programs. Because patients with HIV are at risk for TB throughout life, activities should be ongoing in paediatric and adult ART clinics, pre-ART clinics (keeping relatively healthy patients engaged in care), and maternal health programs

## Conclusion

This study has shown the presence of hot spots of TB-HIV deaths in Africa and continued upward trend in TB-HIV mortality, providing more information on priority areas for public health planning and resource allocation for preventing TB. The study has also demonstrated that using existing health data, GIS and GIS-based spatial statistical techniques could provide an opportunity to clarify and quantify the health burden from TB-HIV deaths within highly endemic areas and also lay a foundation to pursue further investigation into the environmental factors responsible for increased disease risk.

## Competing interests

The authors declare that they have no competing interests.

## Funding

none

## Authors' contributions

OAU: Major role in study conception, data extraction, analyses, and writing of the manuscript. IY: Data extraction, statistical analyses of data and manuscript writing. KA: Data extraction, statistical analyses of data and manuscript writing. MMB: Data extraction, statistical analyses of data and manuscript writing. All authors have read and approved the final manuscript.

## References

[B1] Rieder HL Cauthen GM Comstock GW Snider DE Jr Epidemiology of tuberculosis in the United States Epidemiol Rev 1989 11 79 98 2680563 10.1093/oxfordjournals.epirev.a036046

[B2] Corbett EL Watt CJ Walker N Maher D Williams BG Raviglione MC Dye C The growing burden of tuberculosis: global trends and interactions with the HIV epidemic Archives of internal medicine 2003 163 9 1009 1021 10.1001/archinte.163.9.1009 12742798

[B3] Lawn SD Churchyard G Epidemiology of HIV-associated tuberculosis Curr Opin HIV AIDS 2009 4 4 325 333 10.1097/COH.0b013e32832c7d61 19532072 PMC4233405

[B4] Havlir DV Getahun H Sanne I Nunn P Opportunities and challenges for HIV care in overlapping HIV and TB epidemics Jama 2008 300 4 423 430 10.1001/jama.300.4.423 18647985

[B5] Lawn SD Myer L Bekker LG Wood R Burden of tuberculosis in an antiretroviral treatment programme in sub-Saharan Africa: impact on treatment outcomes and implications for tuberculosis control Aids 2006 20 12 1605 1612 10.1097/01.aids.0000238406.93249.cd 16868441

[B6] Lawn SD Butera ST Shinnick TM Tuberculosis unleashed: the impact of human immunodeficiency virus infection on the host granulomatous response to Mycobacterium tuberculosis Microbes Infect 2002 4 6 635 646 10.1016/S1286-4579(02)01582-4 12048033

[B7] Wilkinson D Davies GR The increasing burden of tuberculosis in rural South Africa--impact of the HIV epidemic S Afr Med J 1997 87 4 447 450 9254788

[B8] Kandala NB Magadi MA Madise NJ An investigation of district spatial variations of childhood diarrhoea and fever morbidity in Malawi Soc Sci Med 2006 62 5 1138 1152 10.1016/j.socscimed.2005.07.028 16139938 PMC7126797

[B9] Tanser FC Le Sueur D The application of geographical information systems to important public health problems in Africa International journal of health geographics 2002 1 1 4 10.1186/1476-072X-1-4 12537589 PMC149399

[B10] World Health Organization Global tuberculosis control. Surveillance, planning and financing. WHO/HTM/TB/2008.393 2008 Geneva: World Health Organization

[B11] Dye C Bassili A Bierrenbach AL Broekmans JF Chadha VK Glaziou P Gopi PG Hosseini M Kim SJ Manissero D Onozaki I Rieder HL Scheele S van Leth F Werf M van der Williams BG Measuring tuberculosis burden, trends, and the impact of control programmes Lancet Infect Dis 2008 18201929 10.1016/S1473-3099(07)70291-8

[B12] Fang L Yan L Liang S de Vlas SJ Feng D Han X Zhao W Xu B Bian L Yang H Gong P Richardus JH Cao W Spatial analysis of hemorrhagic fever with renal syndrome in China BMC Infect Dis 2006 6 77 10.1186/1471-2334-6-77 16638156 PMC1471792

[B13] Kafadar K Smoothing geographical data, particularly rates of disease Stat Med 1996 15 23 2539 2560 10.1002/(SICI)1097-0258(19961215)15:23<2539::AID-SIM379>3.0.CO;2-B 8961462

[B14] Kafadar K Geographic trends in prostate cancer mortality: an application of spatial smoothers and the need for adjustment Ann Epidemiol 1997 7 1 35 45 10.1016/S1047-2797(96)00101-9 9034405

[B15] Smoothing http://www.wiley.com//legacy/wileychi/eoenv/pdf/Vas029-.pdf

[B16] Standardised Mortality Ratios - the effect of smoothing ward-level results http://www.statistics.gov.uk/articles/hsq/HSQ40-Mortality-Ratios.pdf 19093638

[B17] Anselin L Local indicators of spatial association--LISA Geographical Analysis 1995 27 93 115

[B18] Anselin L Sridharan S Gholston S Using Exploratory Spatial Data Analysis to Leverage Social Indicator Databases: The Discovery of Interesting Patterns Social Indicators Research 2007 82 2 287 309 10.1007/s11205-006-9034-x

[B19] Anselin L Syabri I Kho Y GeoDa: An Introduction to Spatial Data Analysis Geographical Analysis 2006 38 1 5 22 10.1111/j.0016-7363.2005.00671.x

[B20] GeoDA 0.9.5-i for Windows http://geodacenter.asu.edu/software/downloads

[B21] Zwang J Garenne M Kahn K Collinson M Tollman SM Trends in mortality from pulmonary tuberculosis and HIV/AIDS co-infection in rural South Africa (Agincourt) Trans R Soc Trop Med Hyg 2007 101 9 893 898 10.1016/j.trstmh.2007.04.023 17597174

[B22] Uthman OA Spatial and temporal variations in incidence of tuberculosis in Africa, 1991 to 2005 World Health Popul 2008 10 2 5 15 18978457 10.12927/whp.2008.19962

[B23] Glynn JR Murray J Bester A Nelson G Shearer S Sonnenberg P Effects of duration of HIV infection and secondary tuberculosis transmission on tuberculosis incidence in the South African gold mines Aids 2008 22 14 1859 1867 10.1097/QAD.0b013e3283097cfa 18753936

[B24] Dye C Scheele S Dolin P Pathania V Raviglione MC Consensus statement. Global burden of tuberculosis: estimated incidence, prevalence, and mortality by country. WHO Global Surveillance and Monitoring Project Jama 1999 282 7 677 686 10.1001/jama.282.7.677 10517722

[B25] Corbett EL Marston B Churchyard GJ De Cock KM Tuberculosis in sub-Saharan Africa: opportunities, challenges, and change in the era of antiretroviral treatment Lancet 2006 367 9514 926 937 10.1016/S0140-6736(06)68383-9 16546541

[B26] Asamoah-Odei E Garcia Calleja JM Boerma JT HIV prevalence and trends in sub-Saharan Africa: no decline and large subregional differences Lancet 2004 364 9428 35 40 10.1016/S0140-6736(04)16587-2 15234854

[B27] Ahmed AB Abubakar I Delpech V Lipman M Boccia D Forde J Antoine D Watson JM The growing impact of HIV infection on the epidemiology of tuberculosis in England and Wales: 1999 2003 Thorax 2007 62 8 672 676 10.1136/thx.2006.072611 17311840 PMC2117286

[B28] Velasco M Castilla V Cervero M Sanz J Condes E Gaspar G Torres R Arranz A Barros C Monereo A The changing pattern of tuberculosis and HIV co-infection in immigrants and Spaniards in the last 20 years HIV Med 2008 9 4 227 233 10.1111/j.1468-1293.2008.00550.x 18366446

[B29] Lawn SD Bekker LG Middelkoop K Myer L Wood R Impact of HIV infection on the epidemiology of tuberculosis in a peri-urban community in South Africa: the need for age-specific interventions Clin Infect Dis 2006 42 7 1040 1047 10.1086/501018 16511773

[B30] World Health Organization Global tuberculosis control: Annual report 2007 2007 Geneva: Switzerland: World Health Organization

[B31] World Health Organization Global tuberculosis control. Epidemiology, strategy, finance. WHO/HTM/TB/2009.411 2009 Geneva: World Health Organization

[B32] Anselin L Longley P, Brooks S, Macmillan B, McDonnell R Exploratory spatial data analysis in a geocomputational environment GeoComputation: A primer 1998 New York: Wiley 77 94

[B33] Sridharan S Tunstall H Lawder R Mitchell R An exploratory spatial data analysis approach to understanding the relationship between deprivation and mortality in Scotland Soc Sci Med 2007 65 9 1942 1952 10.1016/j.socscimed.2007.05.052 17706850

[B34] Exploratory spatial data analysis. NCGIA core curriculum in GIScience http://www.ncgia.ucsb.edu/giscc/units/u128/u128.html

[B35] World Health Organization The Three I's: Intensified Case Finding (ICF), Isoniazid Preventive Therapy (IPT) and TB Infection Control (IC) for people living with HIV. Report of a WHO Joint HIV and TB Department Meeting. Geneva, Switzerland, April 2-4, 2008 2008 Geneva: World Health Organization

